# Severe Neutropenia and Esophagitis Associated With Avelumab for Urothelial Carcinoma: A Case Report

**DOI:** 10.1002/ccr3.73086

**Published:** 2026-07-02

**Authors:** Takayuki Hirano, Takashi Kawahara, Yuta Karibe, Genta Iwamoto, Jun Asano, Shusei Fusayasu, Nobuhiko Mizuno, Kazuhide Makiyama, Hiroji Uemura, Masatoshi Moriyama, Junichi Ohta

**Affiliations:** ^1^ Department of Urology Yokohama Municipal Citizen's Hospital Yokohama Japan; ^2^ Departments of Urology and Renal Transplantation Yokohama City University Medical Center Yokohama Japan; ^3^ Department of Urology Yokohama City University Graduate School of Medicine Yokohama Japan

**Keywords:** adverse effects, avelumab, esophagitis, irAE, neutropenia

## Abstract

Bladder cancer prognosis varies with invasiveness, requiring treatments like surgery or chemotherapy. Immune checkpoint inhibitors, such as Avelumab, are standard for advanced cases but can cause side effects like neutropenia and esophagitis, as seen in a reported case during Avelumab maintenance therapy.

## 1 Introduction

The prognosis of bladder cancer differs greatly depending on whether it is non‐muscle invasive, muscle‐invasive, or metastatic. Endoscopic surgery is the mainstay of treatment for non‐muscle invasive bladder cancer, while muscle‐invasive bladder cancer requires treatment centered on total cystectomy. Chemotherapy, mainly platinum‐based drugs, has been used for many years in recurrent or metastatic bladder cancer after total cystectomy.

Recently, the therapeutic efficacy of immune checkpoint inhibitors (ICIs) has been demonstrated in various types of cancer, and their use in bladder cancer is increasing as a standard treatment for advanced cancer and postoperative adjuvant chemotherapy. Avelumab, an anti‐programmed death‐ligand 1 (PD‐L1) antibody, is one of the ICIs. It has shown efficacy as maintenance therapy after primary chemotherapy including platinum‐based agents for unresectable locally advanced or metastatic urothelial carcinoma and is approved in Japan [1].

With ICIs, immune‐related adverse events (irAEs) are often problematic side effects, presenting a wide variety of symptoms. Among them, skin symptoms, colitis, gastrointestinal symptoms such as diarrhea, liver dysfunction, interstitial pneumonia, and endocrine disorders are widely known. Among gastrointestinal symptoms, however, there are limited reports of esophagitis compared to colitis, pancreatitis, and gastritis. Neutropenia, which is relatively common in general anticancer therapy, is rare with ICIs, and Grade 4 neutropenia is reported to be about 0.14% [2]. We report here a case of a patient with Grade 4 neutropenia and Grade 3 esophagitis during avelumab maintenance therapy. To our knowledge, the simultaneous occurrence of these rare irAEs—Grade 4 neutropenia, esophagitis, and cytokine release syndrome (CRS)—during avelumab maintenance therapy has rarely been reported. The present case is of particular interest because all of these toxicities improved after interleukin‐6 (IL‐6) inhibition with tocilizumab, suggesting a shared cytokine‐mediated mechanism and a potential treatment option for clinicians who encounter such overlapping irAEs.

## 2 Case History

**FIGURE 1:**
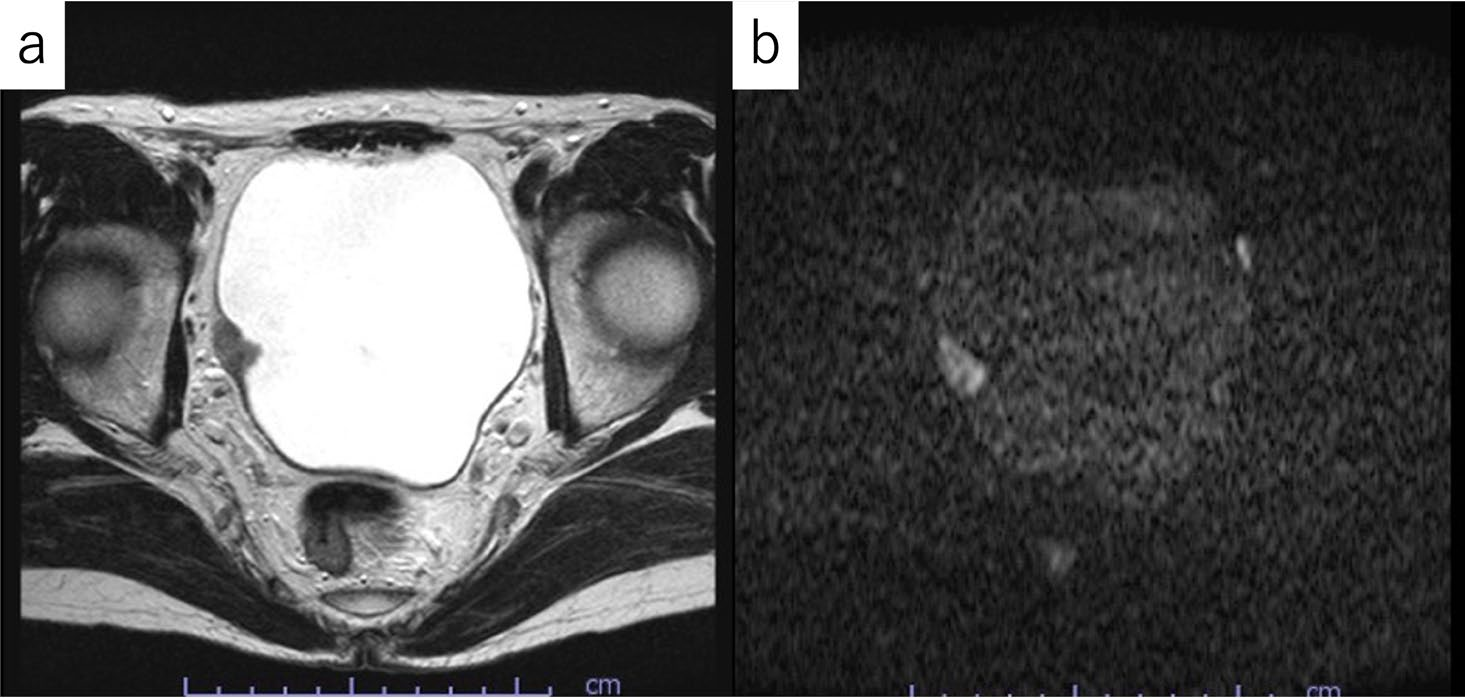
A tumorous lesion with restricted diffusion was observed in the right sidewall.

**FIGURE 2:**
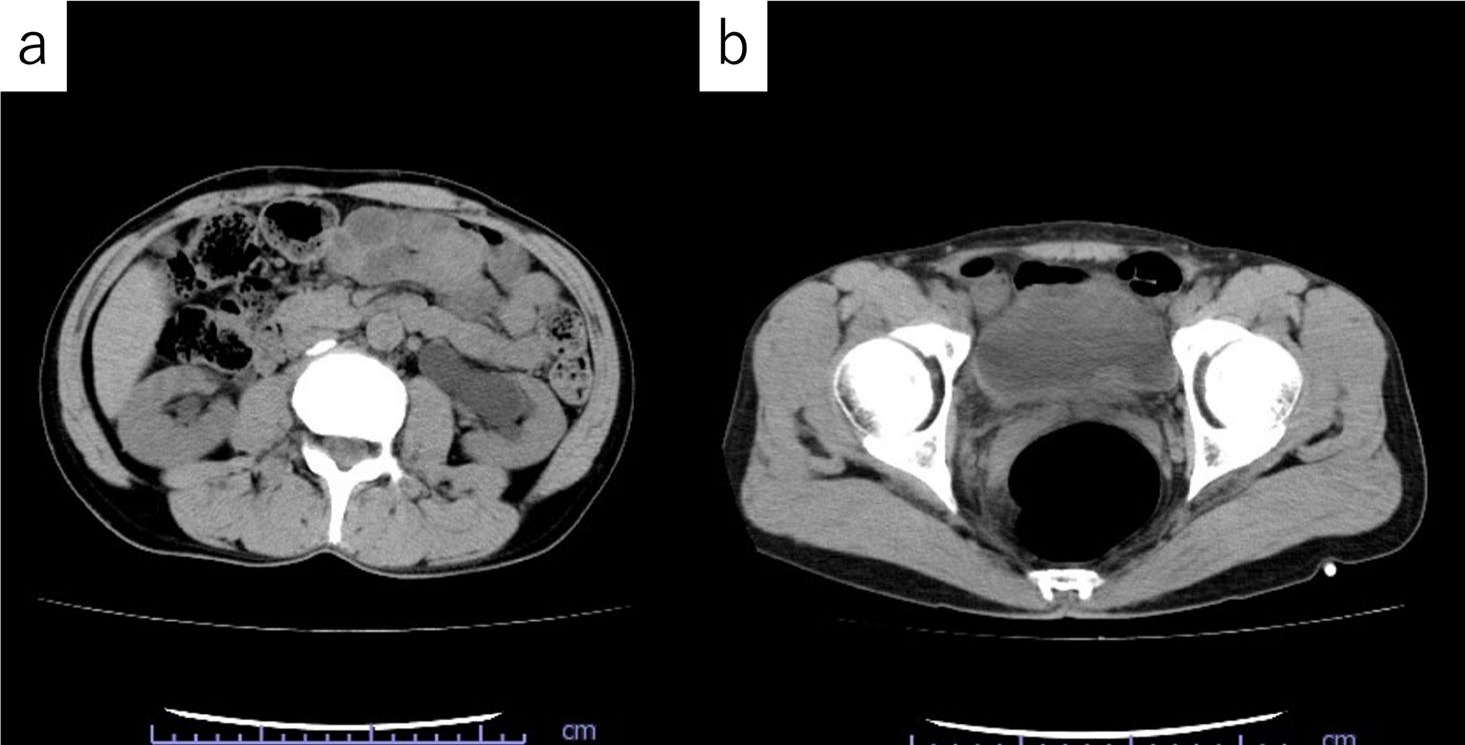
Recurrence of bladder tumor accompanied by left hydronephrosis was identified.

**FIGURE 3:**
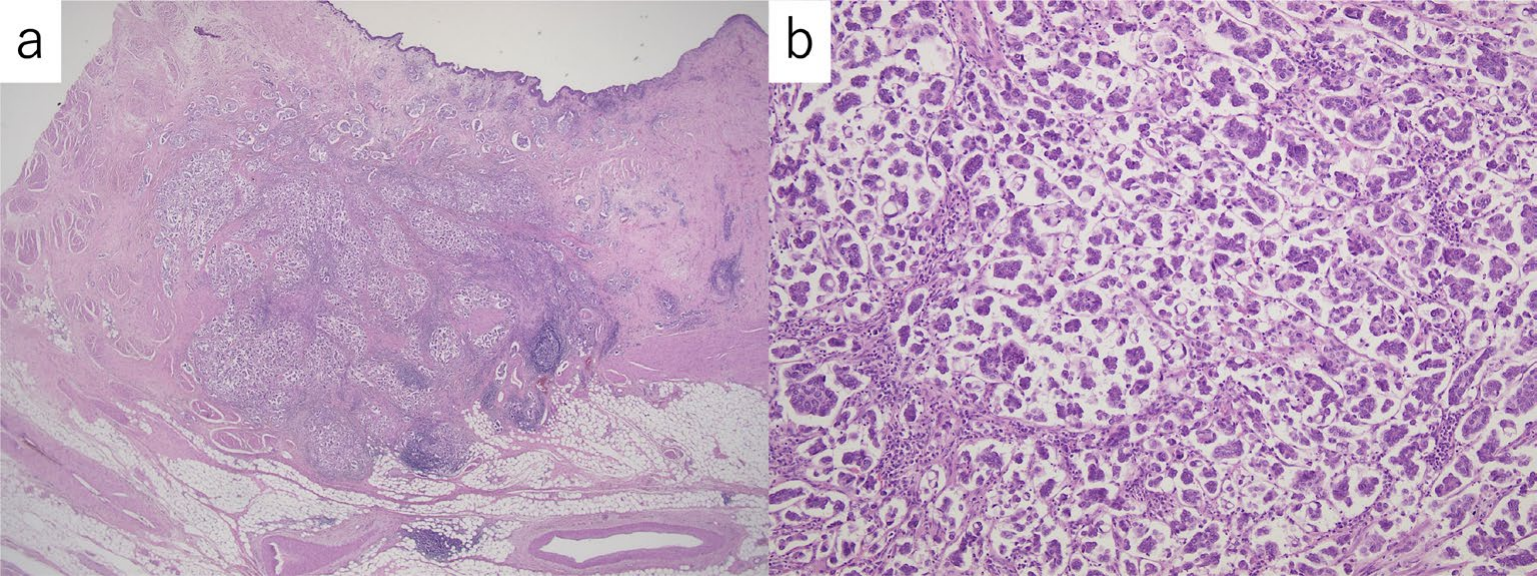
(a) Hematoxylin and eosin (HE) staining weak expansion. (b) Strong expansion, A urinary tract urothelial carcinoma infiltrating up to the fatty layer is observed in weak expansion. In strong expansion, the formation of minute papillary structures is noted. Among the excised lymph nodes, metastasis was observed in 4 out of 16.

In June 20XX‐9, a 54‐year‐old man visited a local urology clinic with a chief complaint of hematuria. Cystoscopy revealed papillary tumors on the posterior wall and right lateral wall of the bladder. He was referred to our department. An initial magnetic resonance imaging (MRI) showed a mass lesion with diffusion restriction on the right lateral wall (Figure 1). A transurethral resection of bladder tumor (TUR‐BT) was performed, and he was diagnosed with urothelial carcinoma T2 or higher. Although a radical cystectomy was proposed, the patient refused. TUR‐BT was repeatedly performed to monitor the recurrences. In January 20XX‐6, a recurrent bladder tumor accompanied by right hydronephrosis was observed (Figure 2), leading to an open radical cystectomy and ileal conduit construction. Pathological results confirmed invasive urothelial carcinoma with micropapillary variant, diagnosed as T3aN2M0 (Figure 3).

### 2.1 Methods (Differential Diagnosis, Investigations and Treatment)

As adjuvant chemotherapy, two courses of gemcitabine and cisplatin (GC) therapy were administered. In November 20XX‐6, computed tomography (CT) scans revealed metastasis to the para‐aortic and iliac lymph nodes, and an additional four courses of GC therapy were conducted. During GC therapy, significant leukopenia was observed. In April 20XX‐5, CT scans showed a tendency for lymph node metastasis to increase. With a diagnosis of progressive disease (PD), pembrolizumab was initiated. In August 20XX‐5, the patient developed Grade 3 diarrhea and was referred to the gastroenterology department, where he was diagnosed with ulcerative colitis caused by an irAE, leading to the discontinuation of pembrolizumab. As the lymph node metastasis remained reduced, the decision was made to follow up with imaging. The patient remained stable without exacerbation. In April 20XX‐1, CT scans again showed an increase in lymph node metastasis. Given the time elapsed since the last administration and the significant leukopenia observed during GC therapy, gemcitabine plus carboplatin (GEM+CBDCA) therapy was administered. After the administration of GEM+CBDCA therapy, lymph nodes reduced, and avelumab maintenance therapy was initiated. Subsequently, during the avelumab maintenance therapy, the reduction in lymph node metastasis was maintained, and the patient continued with the therapy up to the 24th course. On September 21, 20XX, the planned 25th course of avelumab was canceled due to a neutrophil count drop to 410/μL. On October 3, the patient visited due to fever, fatigue, and rash, and was found to have a neutrophil count of 90/μL, hemoglobin (Hb) of 8.7 g/dL, and C‐reactive protein (CRP) of 12.8 mg/dL. Fever, neutropenia, anemia, and rash due to irAE were suspected, and the decision was made to admit the patient for treatment.

### 2.2 Hospital Course

**FIGURE 4:**
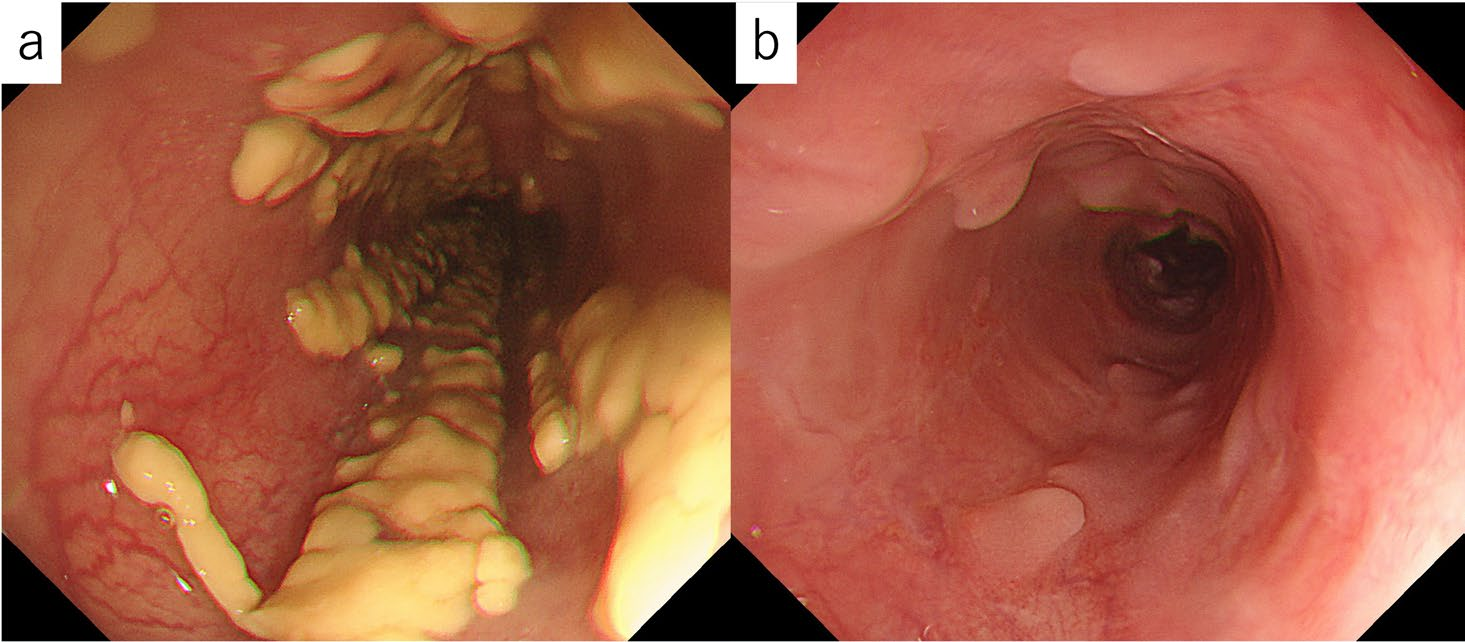
(a) First time upper gastrointestinal endoscopy. Multiple longitudinal ulcers with adherent white exudates are observed. (b) Follow up Upper gastrointestinal endoscopy. Ulcerative lesions with adherent white exudates have disappeared and been replaced by regenerating mucosa.

**FIGURE 5:**
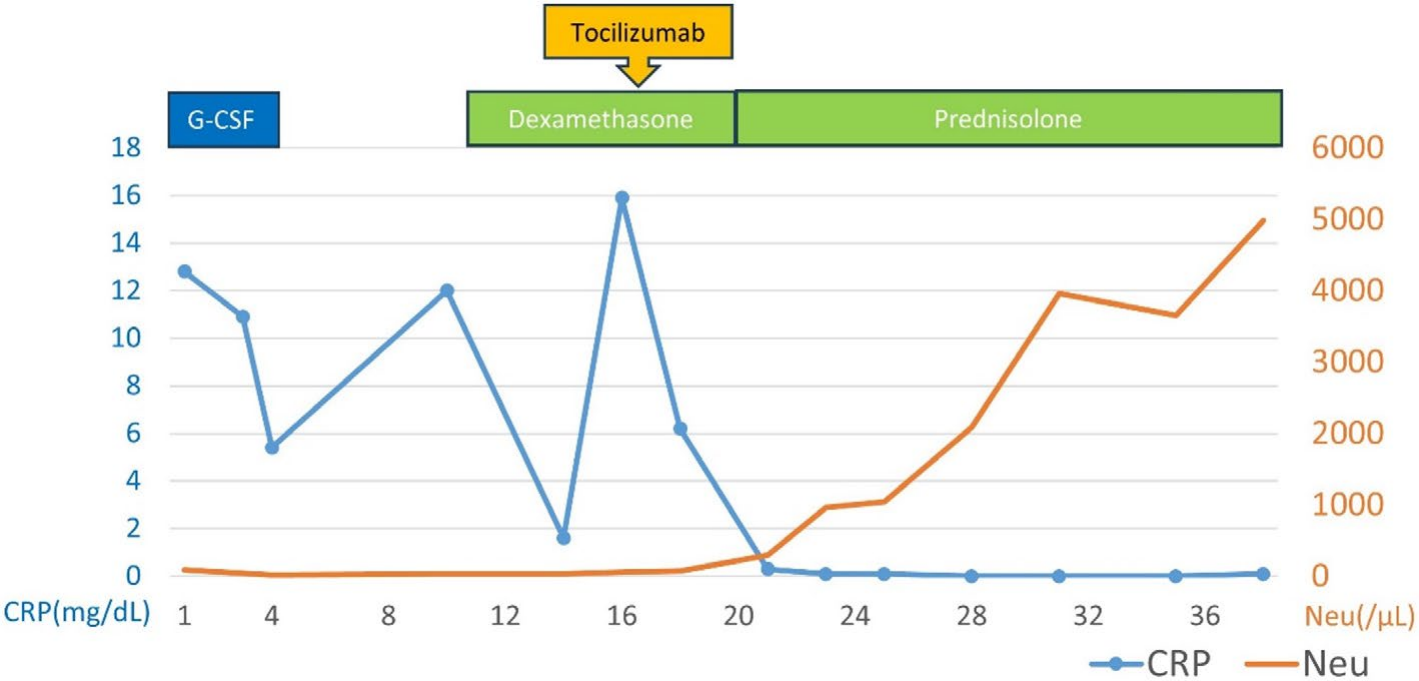
The neutrophil count and CRP during hospitalization fluctuated. The neutrophil count did not improve during G‐CSF and steroid administration, but improvement was observed after administration of tocilizumab.

He was initially treated with granulocyte colony‐stimulating factor (G‐CSF), and antibiotic treatment. But there was no improvement in neutrophil count and fever. After admission, mucosal damage appeared. Despite daily administration of G‐CSF, there was no response, and on the fourth hospital day, the neutrophil count dropped to 20/μL. Considering the risk of infection, G‐CSF was discontinued, and the patient was referred to the hematology department. A bone marrow biopsy was performed on the eighth hospital day, but there were no findings suggestive of a clear hematological disorder. After consultation with both the hematology and respiratory departments, CRS was suspected due to fever, rash, and fatigue, and dexamethasone treatment was initiated. On the eleventh hospital day, the Hb level dropped to 5.6 g/dL, necessitating a red blood cell (RBC) transfusion. After the administration of dexamethasone, the rash showed a tendency to subside, but on the 13th hospital day, symptoms of esophageal pain appeared. Proton pump inhibitors (PPI), sodium alginate, and amphotericin were administered, but the symptoms did not improve, and there was no improvement in neutrophil count either. Elevated levels of soluble interleukin‐2 (IL‐2) (5353 U/mL) and IL‐6 (41.8 pg/mL) were observed, leading to a diagnosis of CRS on the 15th hospital day, and tocilizumab was administered. Additionally, for further examination of the esophageal pain, a gastroenterologist was consulted, and an upper gastrointestinal endoscopy was performed. The endoscopy revealed longitudinal ulcers with white plaques (Figure 4), and given the severity of the clinical course, a diagnosis of irAE esophagitis was made. In consultation with the gastroenterology department, steroid treatment was switched to soluble prednisolone, similar to the treatment for irAE colitis. Due to the severe pain caused by the esophagitis, a continuous subcutaneous injection of fentanyl was started for pain control. Subsequently, the neutrophil count gradually recovered, and the symptoms of esophagitis and food intake improved. A follow‐up upper gastrointestinal endoscopy was performed on the 39th hospital day, which showed improvement in the esophagitis, and the patient was discharged on the 40th hospital day. At an outpatient follow‐up after discharge, blood tests showed an improvement in IL‐6 levels to 10.8 pg/mL (Figure 5).

## 3 Conclusions

In this case, neutropenia, anemia, and esophagitis were suspected to be irAEs, and it is speculated that cytokine storm was ameliorated by IL‐6 inhibition, resulting in improvement of all irAEs.

## 4 Discussion

ICIs provide benefits to the treatment of various cancers.

In urology, there is an increasing opportunity to use ICIs as standard treatments for advanced cancers, as well as for adjuvant chemotherapy. However, ICIs come with the risk of irAEs, which are known to manifest with a variety of symptoms, some of which can be severe or even fatal. IrAEs commonly affect the skin, gastrointestinal tract, liver, lungs, and endocrine system [3]. Conversely, conditions such as CRS, neutropenia, and esophagitis are relatively rare irAEs. This case involved a combination of these rare adverse events.

CRS, known to occur as a result of chimeric antigen receptor T‐cell (CAR‐T) therapy or the use of ICIs, is triggered by the release of large amounts of cytokines from activated T cells or tumor cells. Symptoms include fever, tachypnea, headache, tachycardia, rash, and in severe cases, hypotension and hypoxemia [4]. The incidence after ICIs use is estimated to be around 0.05%–0.14% [5]. Initial symptoms mimic those of a common cold, and in cases involving infection, treatment with antibiotics alongside steroid administration may be necessary. IL‐6 plays a central role in the pathophysiology of CRS [6], and in severe or steroid‐refractory cases, the use of tocilizumab, an anti‐IL‐6 receptor monoclonal antibody, is recommended for suppressing cytokine storms [7]. In this case, the patient presented with cold‐like symptoms and a rash, but without hypotension or hypoxemia. Therefore, initial treatment for CRS involved steroid administration. However, as improvement was insufficient for other symptoms despite improvement in the rash, tocilizumab was administered, resulting in improvement in cold‐like symptoms following administration.

Chemotherapy‐induced bone marrow suppression is a common side effect, whereas a decrease in neutrophil count with ICIs is a rare condition [8]. Treatment typically involves antibiotic therapy and administration of G‐CSF, but if these are ineffective, steroid administration may be considered. In cases where steroids are also ineffective, intravenous immunoglobulin (IVIG) or cyclosporine administration may be considered [9]. Reports have also shown improvement with rituximab administration [10]. In this case, initial treatment included antibiotic therapy and G‐CSF administration, but did not improve. Steroid administration was also ineffective, but improvement in blood cell count was observed following tocilizumab administration. Anemia was also noted in this case, necessitating RBC transfusion. When observing decreases in multiple blood cell lines, consideration should be given to conditions such as immune‐related aplastic anemia or myelodysplastic syndrome [9], although in this case, bone marrow biopsy did not reveal any definitive findings, suggesting immune‐related blood cell reduction.

Compared to colitis, which is commonly observed as a gastrointestinal symptom of irAEs, reports of esophagitis are extremely rare. Reported findings of irAE esophagitis include circumferential shallow ulcers [11], diffuse erythema, mucosal edema [12], and diverse endoscopic findings such as longitudinal ulcers accompanied by white exudates similar to those observed in this case [13]. Diagnosis based solely on endoscopic findings may be difficult, and it is necessary to consider the clinical course in conjunction with endoscopic findings. Although no established treatment exists, there have been reports of improvement with prednisolone administration similar to irAE colitis [11]. In this case, tocilizumab was administered due to elevated levels of IL‐6, which resulted in improvement, as reported in other cases [12]. Retrospective studies have also reported symptom improvement in patients with various irAEs following tocilizumab administration [14]. In cases where steroid therapy is ineffective and there is a high concentration of IL‐6 in the blood, consideration should be given to administering tocilizumab.

## Author Contributions

**Takayuki Hirano:** conceptualization, data curation, writing – original draft. **Takashi Kawahara:** conceptualization, methodology, writing – original draft. **Yuta Karibe:** data curation. **Genta Iwamoto:** data curation, methodology. **Jun Asano:** data curation. **Shusei Fusayasu:** data curation. **Nobuhiko Mizuno:** data curation. **Kazuhide Makiyama:** supervision. **Hiroji Uemura:** data curation, supervision, validation. **Masatoshi Moriyama:** supervision. **Junichi Ohta:** methodology, supervision.

## Funding

The authors have nothing to report.

## Ethics Statement

The authors have nothing to report.

## Consent

Written informed consent was obtained from the patient for publication of this case report and any accompanying images. A copy of the written consent is available for review by the Editor‐in‐Chief of this journal.

## Conflicts of Interest

The authors declare no conflicts of interest.

## Data Availability

The authors have nothing to report.
